# Spina Bifida Occulta of the Sacrum as a Contributing Factor to Progressive Scoliosis

**DOI:** 10.7759/cureus.112712

**Published:** 2026-07-15

**Authors:** Eric Chun-Pu Chu

**Affiliations:** 1 Chiropractic and Physiotherapy Center, New York Medical Group, Hong Kong, CHN

**Keywords:** chiropractic, ligamenta flava, maternal folic acid, sacrum, scoliosis, spina bifida occulta

## Abstract

Spina bifida occulta (SBO) refers to a split in the spine that is concealed beneath the skin. The biomechanical impact of complete sacral SBO has often been underappreciated. This case highlights the potential effects of complete sacral SBO on the spine. A 12-year-old girl presented with truncal asymmetry and intermittent neck and lumbar pain lasting for two years. Initially, she was diagnosed with mild scoliosis during a school health screening and was placed under medical observation, but her symptoms worsened over time. Imaging studies revealed an inverted cervical lordosis along with a 25-degree left thoracolumbar curvature. Additionally, the laminae of all five sacral vertebrae were intact yet failed to fuse into a median crest, consistent with a diagnosis of complete sacral SBO. This anomaly was not noted in the patient's earlier medical documentation. The treatment aimed to improve spinal stability. Chiropractic care was provided weekly for 58 months, incorporating rhythmic massage, flexion-distraction techniques, and spinal manipulation. The patient reported complete resolution of neck and back pain, and her scoliotic curve was reduced to 17 degrees. This case underscores the biomechanical significance of sacral SBO in lumbosacral stability, an issue that has largely been overlooked in clinical research.

## Introduction

Spina bifida occulta (SBO) is a condition in which vertebral laminae fail to fuse at various segments of the spine without damaging the spinal cord or meninges. The S1 segment has the highest prevalence of openings, but non-fusion rarely extends from S1 to S5, resulting in complete sacral SBO [1-3]. SBO is probably underreported as it can be symptomless and often ignored. Anatomical studies reported prevalence rates of 1.3% (4 out of 300) [4] and 4.5% (9 out of 200) [5] in dry sacral specimens. In most clinical settings, SBOs are regarded as anatomical variations and are generally disregarded on routine radiographs in asymptomatic individuals [5].

When the midline posterior sacral architecture is un-united, it does not directly compromise the vertical load-bearing stability of the solid sacral base; instead, it alters regional kinetic distribution by modifying the attachment pathways and continuous tension lines of the regional paraspinal musculature and the dense layers of the thoracolumbar fascia [6]. The median sacral crest serves as a key insertion site for these fascial planes, which play a critical role in transferring multi-planar asymmetric forces between the pelvis and the mobile spinal column above [7]. A delay or failure in this posterior midline fusion can disrupt the symmetry of these fascial tension networks, creating an imbalance in the paraspinal muscles that acts as a permissive biomechanical factor for the development of chronic spinal pain and progressive adolescent scoliosis [6]. Furthermore, an extensive failure of the sacral laminae to fuse leaves the sacral canal posteriorly unprotected by bone, which may increase the vulnerability of the underlying sacral nerve roots to external mechanical pressure or localized structural traction [8]. 

This case study highlights the biomechanical impacts of complete sacral SBO, an issue that has largely been neglected in clinical practice. Improving spinal biomechanics can often restore stability and reduce related discomfort. The author delves into the connection between scoliosis and sacral SBO, shedding light on the pathophysiological challenges associated with sacral SBO in the context of developing spines.

## Case presentation

A 12-year-old girl came in with truncal asymmetry and occasional neck and lower back pain that had persisted for two years. The patient reported no history of prior injuries, neurological issues, or other concerning symptoms. Pain was rated 4 out of 10 on the numeric pain scale. A year prior to this referral, she was identified as having mild scoliosis during a health check at school. Her health records indicated a 19° left thoracolumbar curve, a 9° right thoracic curve, and a Risser 0 bone maturity level at that time. The patient received consistent medical monitoring as she continued to grow, with a mild scoliosis curve measuring less than 25° in Cobb angle [9]. However, both the neck and back pain intensified, and the curvature became more pronounced throughout the observation period. Subsequently, her mother took her to see a chiropractor for evaluation and potential treatment of her symptoms.

**Figure 1:**
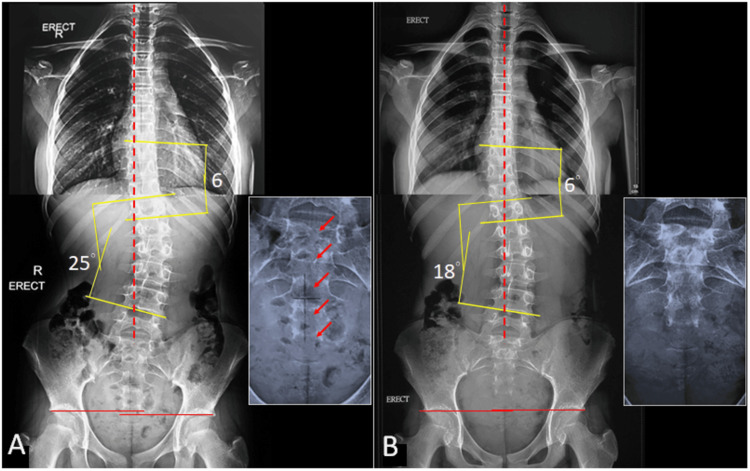
Radiographs comparing the spinal alignment on standing view. (A) At initial presentation, radiographs show a left thoracolumbar curve between T12-L4 of 25° Cobb angle and a right compensatory thoracic curve between T9-T12 with 6° Cobb angle. The Risser sign is 1. The posterior laminae of the sacral segments remained unfused without a distinct median crest. While complete sacral consolidation is an ongoing developmental process that continues into early adulthood, this prominent lack of midline fusion at 12 years of age represents a delayed or incomplete posterior arch ossification pattern, signaling an underlying lumbosacral structural variation. The red arrows indicate the edges of the open dorsal arch of the sacral crest. (B) At the eighth-month evaluation, the major curve has reduced by 7° (from Cobb 25° corrected to 18°) as compared to the angles on initial radiographs. The Risser sign is 1. The central sacral vertical line (dashed red line) represents the global axis.

**Figure 2:**
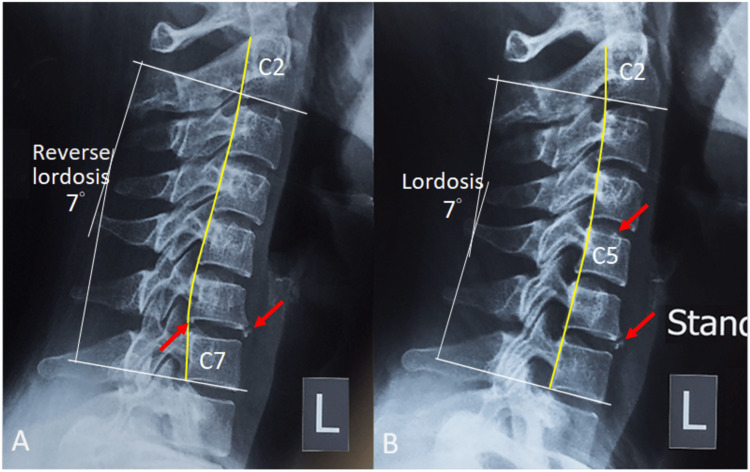
Radiographs illustrating the cervical alignment in lateral view. (A) Lateral radiograph taken during the patient's initial presentation-previously detailed in Figure 1-reveals reverse cervical lordosis (yellow line highlights the posterior vertebral body margins). (B) A radiograph obtained at the eighth-month follow-up shows the restoration of cervical lordosis.

At the time of presentation, the patient was a healthy school-girl weighing 45 kg and measuring 160 cm in height. She was 11 months post-menarche. Asymmetry was noted at the shoulder and waist areas. The Adam's forward bend test confirmed the presence of a scoliotic deformity with a right-sided rib hump. No differences in leg length were detected clinically. Radiographic images (Figures 1, 2) revealed a reversed cervical lordosis, a left thoracolumbar curve from T12 to L4 with a Cobb angle of 25°, and a right compensatory thoracic curve from T9 to T12 with a Cobb angle of 6°. The Risser sign was grade 1. Additionally, there was non-fusion of the posterior laminae of the S1 to S5 vertebrae. Her condition was described as a progressive thoracolumbar scoliosis concurrent with an incomplete posterior midline fusion of the sacrum, with her primary lumbar curve measuring 25°.

The patient's medical history was unremarkable for major trauma, metabolic bone disease, or connective tissue disorders. Furthermore, a comprehensive three-generation genetic screening revealed no family history of scoliosis, spinal deformities, or early-onset spinal pathologies on either the maternal or paternal sides, suggesting a sporadic presentation.

To address the patient's acute discomfort and establish structural tolerance, an initial high-frequency care phase was implemented. The treatment involved a 10-minute rhythmic massage performed with a G5® machine (General Physiotherapy, Inc., St. Louis, Missouri, USA). This was followed by flexion distraction therapy, during which the patient lay face down on a motorized table. The caudal section of the table was alternately raised and lowered, creating intermittent distraction of the vertebral segments [9]. A total of five treatment sessions were scheduled over the course of one consecutive week, administered daily from Monday through Friday with no intervening rest days, followed by a two-day weekend recovery period. The intensity of these early rehabilitation and treatment sessions was maintained at a low-to-moderate, sub-threshold level to accommodate the patient's adolescent skeletal maturity. This approach integrated gentle, low-force rhythmic soft-tissue mobilization and mechanically assisted flexion-distraction tables, carefully avoiding high-force manual manipulations or strenuous resistive loading that could provoke muscular guarding or increase joint strain.

The patient reported noticeable improvement within the first week of treatment. The therapy protocol using a previously published scoliosis correction method developed and validated by our group, remained consistent during the second phase, incorporating high-force, low-amplitude spinal manipulations [9]. A targeted thrust was applied to the apex (L2) of the curve during a 10-minute session of flexion distraction [9]. Subsequently, the treatment frequency was adjusted to three sessions per week over a six-month period. By the end of this phase, the patient experienced complete relief from pain and regained full mobility in both the neck and back. Enhanced physical functionality was attributed to measurable changes observed in follow-up radiographs taken eight months into treatment. These images confirmed correction of the patient's straight neck and a significant reduction in the primary curve, with the Cobb angle improving from 25° to 18° (Figures 1B, 2B).

Subsequently, the patient followed a weekly maintenance program over the next four years, incorporating scoliosis correction techniques, symptom monitoring, home-based stretching exercises, and ergonomic adjustments. The patient was prescribed specific, brief ergonomic adjustments to minimize asymmetric spinal loading during daily activities. These modifications included: (1) an asymmetric sitting posture protocol using a tailored pelvic wedge support to un-weight her left-sided curve convexity at her school desk; (2) a strict load-distribution rule requiring a symmetrical, dual-strap backpack maintained at less than 10% of her body weight; and (3) digital device height optimization to keep reading materials at eye level, thereby eliminating prolonged forward head flexion and mitigating adverse mechanical tension across the lumbosacral and thoracic regions.

**Figure 3:**
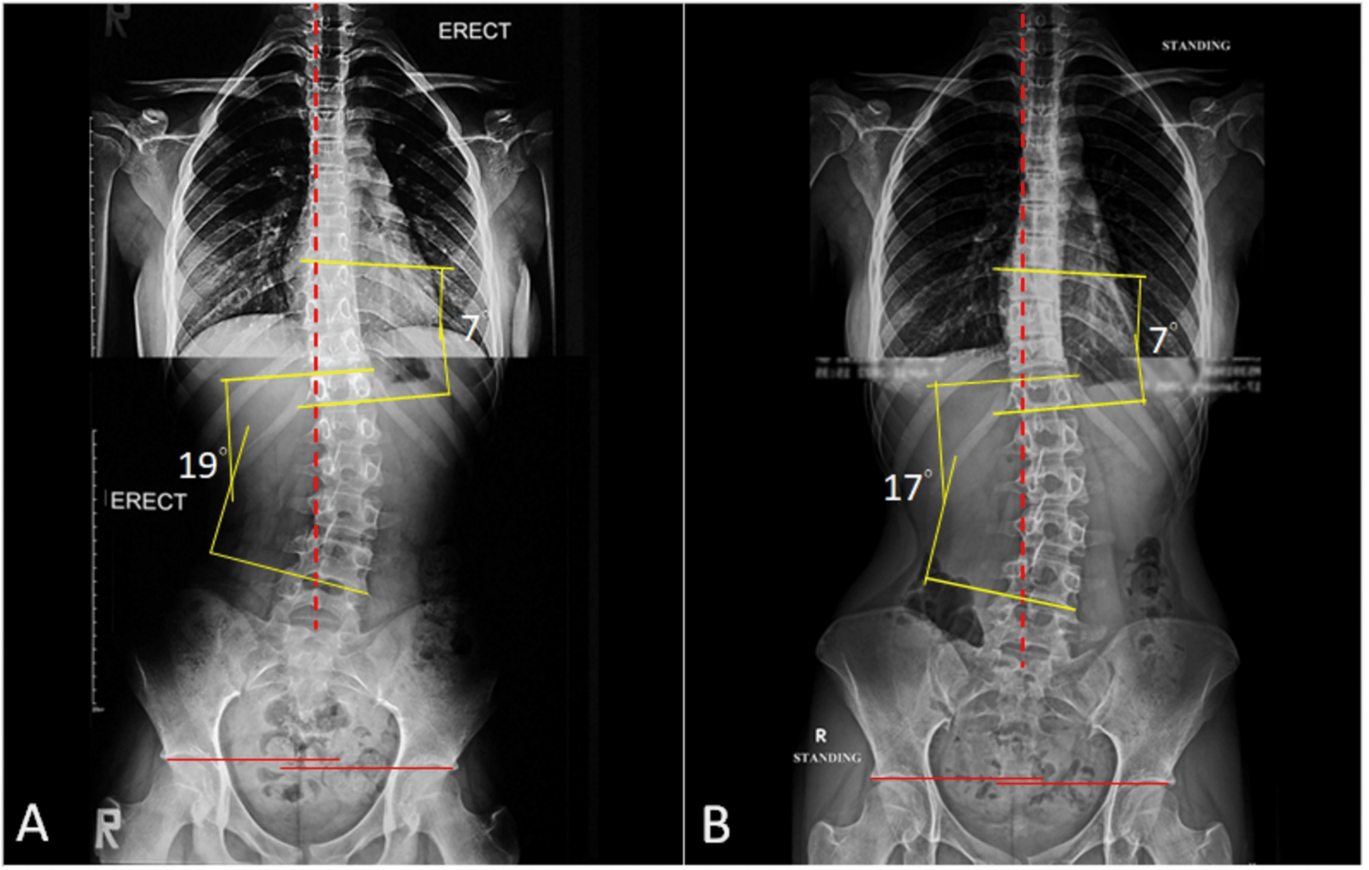
A comparison of treatment outcomes for the patient over time, highlighting progressive structural changes. (A) Fourteen months following the start of treatment, the coronal Cobb angle of the primary thoracolumbar curve measures 19°, while the compensatory thoracic curve is 7°. The Risser sign is classified as level 1. (B) Long-term follow-up radiography demonstrating progressive sacral consolidation. Repeat radiographs taken 58 months after treatment initiation demonstrate sustained correction. The thoracolumbar curve is measured at 17°, and the compensatory thoracic curve remains at 7°, with the Risser sign now observed at 4.

Fourteen months after initiating treatment, the coronal Cobb angle of the primary thoracolumbar curve decreased to 19°, whereas the compensatory thoracic curve measured 7° (see Figure 3A). At the final follow-up, conducted during the 58th month, the corrections remained stable. The thoracolumbar curve was recorded at 17°, with the compensatory thoracic curve unchanged at 7°. The Risser sign indicated a score of 4 (refer to Figure 3B). Factors contributing to discontinuation of additional manipulative therapy included the patient's age (≥17 years), her being more than five years post-menarche, and nearing skeletal maturity (Risser 4 level). The patient (now 17 years old) demonstrated healthy physical development, with her weight increasing to 52 kg and her height reaching 166 cm. Patient reported no more symptoms. Both the patient and her parents expressed satisfaction with the results. No neurological issues or significant complications associated with the treatment were observed.

## Discussion

The embryological and pediatric development of the sacrum involves a prolonged ossification timeline, where the posterior laminae of the sacral segments gradually fuse along the midline, a process that frequently continues into early adulthood [10]. When this midline osseous consolidation is chronologically delayed or protracted during adolescence, the structural continuity of the posterior sacral elements is temporarily altered [11]. Rather than representing an open neural tube defect or permanent congenital malformation, these delayed ossification windows can influence the anchor points of the deep paraspinal musculature and thoracolumbar fascia, occasionally modifying regional kinetic pathways during rapid skeletal growth.

Spina bifida occulta (SBO) represents a concealed form of spina bifida where the vertebral defect remains covered by a layer of skin. In an investigation of 1,061 Chinese adults with a mean age of 58.8 years, Wu et al. [12] found that 14.6% (155/1,061) had sacral SBO. Another study conducted by Li et al. focused on 148 younger Chinese individuals with a median age of 23 years, all presenting with lower back pain [1]. This study reported a lumbosacral SBO prevalence of 62.8% (93/148) [13]. Evidence suggests a diminishing prevalence of sacral SBO within older populations, due to the ossification of cartilaginous vertebral arches as vertebrae mature into adulthood. Complete sacral SBO, wherein all sacral arches from S1 to S5 fail to fuse, is considered exceptionally rare among sacral malformations [2,6].

While the posterior elements of the sacrum are not primary direct load-bearing stabilizers of the lumbosacral junction, an extensive lack of midline fusion can subtly influence regional loading dynamics [14]. The median sacral crest serves as an essential anatomical anchor for the deep paraspinal musculature and the dense bands of the thoracolumbar fascia. An incomplete or delayed midline fusion alters the structural continuity of these fascial and muscular insertions [15]. During rapid adolescent growth spurts, this alteration can lead to asymmetric soft-tissue tension vectors across the base of the spine, which may act as a permissive mechanical factor for the progression of an existing scoliotic curve above it [16,17]. Scoliosis is present in approximately half of patients with spina bifida [18]. Posteriorly, sacral SBO can compromise the sacroiliac joints and increase the risk of pelvic ring instability [3].

In scoliosis, significant anatomical changes occur within the soft-tissue structures supporting the vertebral bodies [16]. One proposed mechanism describes that the scoliotic spine employs a balancing attempt to achieve equilibrium. Depending on the outcome of this rebalancing effort, the spinal curve may either stabilize or regress; if unsuccessful, the curve will continue to progress [19]. With its inherent flexibility, adaptability, and responsiveness, the growing spine is particularly suited for early corrective interventions. One study has suggested that manipulative therapy can help slow curve progression and, in some cases, improve the curves associated with adolescent idiopathic scoliosis [20]. Nevertheless, the relationship between scoliosis progression and sacral spina bifida occulta (SBO) remains poorly understood, complicating treatment strategies due to the varied nature of underlying pathologies seen in adolescents with scoliosis [21].

Assessing both curve flexibility and bone maturity is crucial to avoid unnecessary and prolonged treatments in adolescents. According to guidelines established by the International Scientific Society for Scoliosis Orthopaedic and Rehabilitation Treatment, successful outcomes of conservative management are defined as either preventing curve progression of ≤5° (stabilization) or achieving a reduction of ≥6° (correction) from baseline measurements [22]. In this case involving multimodal intervention, follow-up assessment conducted 58 months post-treatment showed a reduction in the major curve from 25° to 17° (an 8° correction). The compensatory curve remained stable, indicating that treatment was successful by clinical standards.

## Conclusions

Developmental malformations are common, with the sacrum representing the most variable section of the spine. Complete sacral SBO is often asymptomatic and seldom identified during diagnostic evaluations. Generally regarded as a minor variant, it is frequently overlooked by radiologists when reviewing lumbosacral radiographs. It is recommended that attending physicians personally assess radiographs to ensure that any sacral abnormalities are recognized and taken into account for effective management of back-related symptoms.
